# Treating cancer cachexia to treat cancer

**DOI:** 10.1186/2044-5040-1-2

**Published:** 2011-01-24

**Authors:** Se-Jin Lee, David J Glass

**Affiliations:** 1 Johns Hopkins University School of Medicine, Department of Molecular Biology and Genetics, 725 N. Wolfe St., PCTB 803, Baltimore, Maryland 21205, USA; 2 Novartis Institutes for Biomedical Research, 100 Technology Square, Cambridge, Massachusetts 02139, USA

## Abstract

Skeletal muscle wasting is a major component of cachectic states found in a variety of disease settings, including cancer. As increasing caloric intake often provides little benefit in combating muscle loss in cachectic patients, a major research focus has been to develop strategies stimulating muscle anabolic pathways - in an attempt to fight the catabolic pathways induced during cachexia. Two recent papers have reported the beneficial effects of blocking the myostatin/activin signalling pathway in mouse models of cancer cachexia. We discuss the implications of their findings both with respect to the role that this signalling pathway may play in the aetiology of cachexia and with respect to the prospects for targeting this pathway as a therapeutic strategy in patients with cachexia.

## Background

Loss of skeletal muscle mass can occur in a wide range of disease states and has significant consequences, causing debilitating weakness and also metabolic dysfunction - as skeletal muscle is one of the major tissues in the body responsible for regulating energy availability and energy expenditure. Loss of muscle mass can result either from primary muscle degenerative diseases, such as the muscular dystrophies, or as a secondary consequence of cachectic disease processes also affecting other tissues, such as cancer, burns, renal failure, sepsis and congestive heart failure (for review, see [1]). In the latter class of conditions, muscle wasting is seen in many patients with various distinct types of cancer. In fact, unexplained weight loss and fatigue are often the presenting symptoms that bring cancer patients to the doctor's office. Moreover, this wasting process, or cachexia, has been cited as a major cause of actual mortality in patients with cancer. The aetiology of cachexia has remained largely mysterious, although several cytokines, including tumour necrosis factor-α, interleukin-6 and interleukin-1β, have been implicated as playing a role in mediating the wasting process. One of the hallmarks of cachexia is that the loss of lean body mass cannot be prevented or reversed simply by increasing nutritional intake. Therefore, there has been considerable focus on developing anabolic strategies that directly target muscle in order to preserve muscle mass and function. Two recent papers by Benny-Klimek *et al*. [2] and Zhou *et al*. [3] have reported studies that investigated the potential beneficial effects of targeting the myostatin/activin signalling pathway in order to provide such an anabolic stimulus to muscle in rodent models of cancer cachexia.

## Discussion

Myostatin (MSTN) is a transforming growth factor-β (TGF-β) family member that normally acts to limit muscle mass (for review, see [4]). Mutations in the *Mstn *gene have been shown to result in dramatic increases in muscle mass in multiple species [5-11], and inhibitors of MSTN signalling have been shown to cause increases in muscle growth when administered systemically to adult mice [12-15]. As a result, there have been extensive efforts directed at developing strategies and agents capable of modulating this signalling pathway for applications in a wide range of clinical settings. The finding that overexpression of MSTN in mice could lead to the development of a cachexia-like syndrome characterized by an extensive loss of fat and muscle [16] raised two important questions about potential therapeutic applications of targeting this pathway in patients with cachexia. First, does the inappropriate activation of this pathway play a causative role in the development of cachexia in humans? Second, whether or not this signalling pathway is involved in the aetiology of cachexia, can blocking this pathway to preserve muscle mass thereby reduce morbidity and mortality in patients with cachexia?

**Figure 1:**
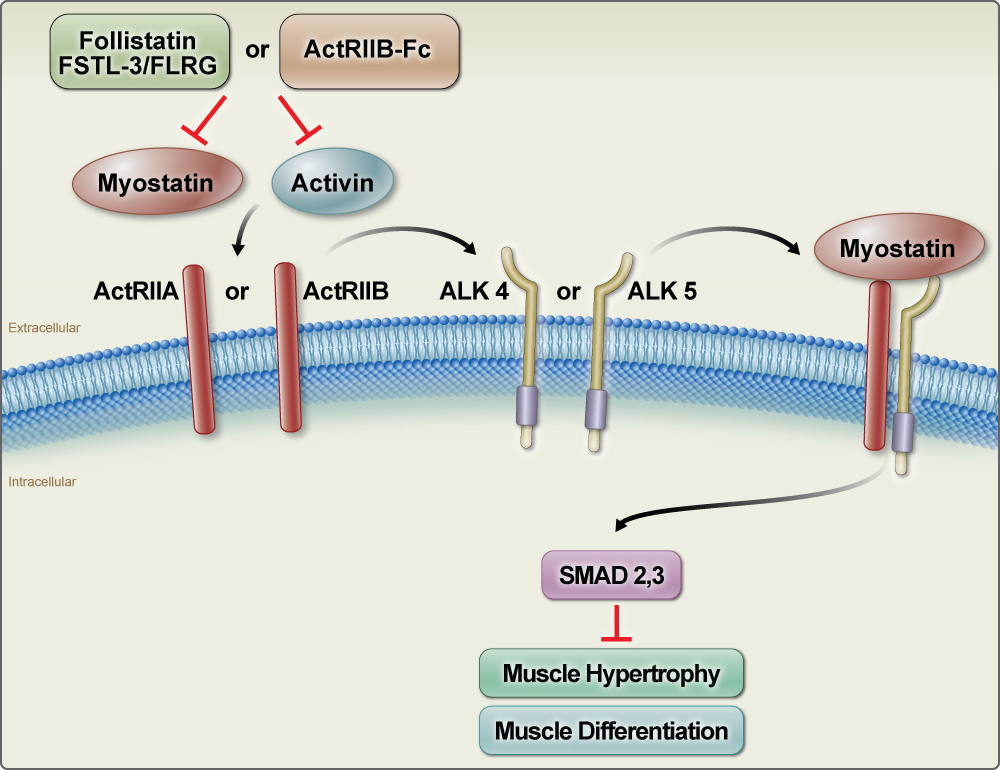
**Inhibition of myostatin (MSTN) and activin signalling by the soluble activin type IIB receptor (ActRIB)**. MSTN and activin signal to target cells by binding initially to the two activin type II receptors, ActRIIA and/or ActRIIB (also called Acvr2 and Acvr2b, respectively) and then to the type I receptors, ALK4 and/or ALK5. The activated type I receptors phosphorylate the intracellular mediators of signalling, Smad2 and/or Smad3. Signalling through this pathway results in the inhibition of muscle differentiation and growth. The activities of MSTN and activin are regulated normally by a number of different extracellular binding proteins, such as follistatin and FSTL-3. The soluble form of ActRIIB (ActRIIB/Fc) can act as a ligand trap by binding MSTN and activin and preventing the ligands from binding to their true receptors.

Benny-Klimek *et al*. [2] and Zhou *et al*. [3] examined the effect of blocking the MSTN pathway in mice bearing cachexia-inducing tumours. Previous studies had shown that MSTN signals by binding initially to activin type II receptors [17] and that a soluble form of the activin type IIB receptor (ActRIIB or ACVR2B), consisting of its ligand binding domain fused to an immunoglobulin Fc domain, can inhibit signalling of MSTN and other TGF-β family secreted proteins that signal via the ActRII receptors, such as the activins [15]. Moreover, the soluble receptor had been shown to cause significant muscle growth (40%-60% in just 2 weeks) when administered systemically to adult mice [15], presumably by acting as a 'trap' of the circulating ligands, binding them in serum and, thereby, preventing binding and activation of the cellular receptor complexes (Figure 1).

Either by inoculating mice with Chinese hamster ovary cells expressing this soluble receptor [2] or by injecting mice directly with the purified fusion protein [3], the two groups showed that blocking this signalling pathway was effective in preserving muscle mass in a wide range of muscle groups, as well as in maintaining forelimb grip strength in mice bearing various tumour cells known to induce wasting. Interestingly, this protective effect was not observed when a different pharmacological agent was used to block this pathway, namely, the deacetylase inhibitor, trichostatin A (TSA). Although previous studies had shown that TSA could increase the expression of the MSTN antagonist, follistatin [18,19], Benny-Klimek *et al*. found that treatment of tumour-bearing mice with TSA could not prevent muscle loss even at doses capable of inducing muscle growth in normal mice. In addition to these effects on skeletal muscle, Zhou *et al*. reported that treatment with the soluble receptor could also prevent cardiac muscle atrophy in tumour-bearing mice. This finding is particularly significant, as there have been concerns that blocking MSTN signalling for clinical applications might have adverse effects on cardiac function. Perhaps the most spectacular result was the finding by Zhou *et al*. that the soluble receptor was capable of increasing survival of mice inoculated with colon-26 (C26) carcinoma cells even though the intervention had no effect on actual tumour growth. Hence, these studies have provided exciting and compelling data that blocking muscle wasting *per se *can have significant beneficial effects on both morbidity and mortality in the setting of cancer and that agents capable of blocking this signalling pathway may be effective means of achieving this end.

What is less clear is whether these studies get us any closer to understanding the role that this signalling pathway may play in the aetiology of cachexia. The fact that muscle mass is preserved by blocking this pathway does not necessarily mean that overactivity of the pathway is responsible for inducing wasting. Blocking MSTN signalling with agents like the soluble receptor is known to induce significant muscle growth, and it could be that these anabolic effects may simply be compensating for the muscle wasting that is being induced by activation of other pathways. In this respect, Zhou *et al*. found *Mstn *messenger RNA (mRNA) levels in muscle to be elevated by about two-fold in C26 tumour-bearing mice. However, Benny-Klimek *et al*. showed using two different tumour lines (Lewis lung carcinoma and B16F10 melanoma) that *Mstn *knockout mice are not only susceptible to tumour-induced wasting but, for reasons that are unclear, actually appear to exhibit an exaggerated response. Zhou *et al*. further examined the role of this signalling pathway by focusing on the possibility that the culprit in cancer cachexia may not be MSTN itself but activins, which are TGF-β family members capable of signalling through the same receptors as MSTN (for review, see [20]). Previous studies had shown that several TGF-β family members, including activins, are as active as MSTN in inhibiting myoblast differentiation, acting through the ActRIIB pathway [21], and that electroporation of an activin A expression cassette directly into muscle can induce myofiber atrophy [22], suggesting that activin A and MSTN may be capable of activating the same signalling cascade leading to wasting. Zhou *et al*. present two sets of studies examining the possible role that activins may play in inducing cancer cachexia.

In one set of studies, they utilized a genetic model of tumourigenesis in which the normal balance of inhibin/activin signalling had been disrupted by a targeted mutation in the *Inha *gene, which encodes the inhibin-α subunit. Inhibins and activins, which generally have counteracting biological activities, are dimers that differ with respect to their subunit composition, with inhibins consisting of α and β subunits and activins consisting of just β subunits. Hence, mice lacking inhibin-α have excess levels of activin signalling, and previous studies have demonstrated that these mice develop both gonadal and adrenal tumours and exhibit a cachexia-like syndrome characterized by severe weight loss, hepatocellular necrosis and gastric mucosal atrophy [23,24]. Zhou *et al*. show that these mice also develop skeletal muscle atrophy and that this muscle wasting can be blocked by administering the soluble activin type IIB receptor. Although these studies demonstrate that excess activin activity can ultimately lead to muscle loss, additional studies are needed in order to help us to understand the relevance of these findings to what may be happening in cancer cachexia. In particular, it will be important to determine the extent to which the effects on muscle seen in this model reflect excess signalling of activin directly to muscle versus activation of atrophy-inducing pathways as a result of tumour development. The interpretation of these studies is somewhat complicated by the fact that the development of tumours in these mice is itself dependent on activin signalling; that is, blocking activin activity using a soluble form of a different activin type II receptor had previously been shown to block not only the cachexia-like syndrome in these mice but also tumour progression [25].

In a second set of studies, Zhou *et al*. surveyed a number of human tumours and identified several that express high levels of activin A. They went on to show that two of these tumour lines could induce muscle loss when inoculated into nude mice and that this wasting process could, again, be blocked by administering the soluble receptor. Although these findings were consistent with the model that increased activin signalling played a causative role in inducing wasting, it will be important to carry out additional studies to further characterize these human tumours with respect to their ability to induce cachexia. For example, is there is correlation between expression levels of activin in the tumour lines and their ability to induce wasting? Similarly, does blocking activin expression in a given tumour line abrogate its ability to induce wasting? Furthermore, as in the case of the tumours that developed in the inhibin-α knockout mice, Zhou *et al*. reported that the soluble receptor also suppressed the growth of the human tumours in mice, making it difficult to attribute the wasting process to the direct effects of activin on muscle.

## Conclusions

What we are left with is the fundamental observation that blocking the MSTN/activin signalling pathway in the context of cancer cachexia can have significant beneficial effects on both morbidity and mortality, leaving somewhat open the question of whether activation of this pathway may play a causative role in this process. This issue of causality could have important implications not only for the understanding of the basic biology of cachexia but also for pursuing this therapeutic strategy to treat cachectic patients. It seems reasonable to expect that the aetiology of cachexia may be complex and/or heterogeneous and may result from aberrant activity of multiple signalling pathways, particularly if one also considers cachexia that results not only from cancer but also from a variety of other disease processes. Hence, it is certainly possible that the MSTN/activin pathway may be activated in only a subset of patients and that the extent to which a given patient's cachexia is responsive or refractory to this therapeutic approach may be dependent on the extent to which the pathway is activated in any individual case.

A causal link between skeletal muscle wasting and activation of MSTN signalling has, perhaps, been more convincingly established in the cachexia seen in the setting of heart failure. In particular, *Mstn *expression has been shown to be upregulated in the heart in animal models of ischemic and pressure overload injury [26-28] as well as in humans with heart failure [29]. Importantly, mice carrying a heart-specific knockout of the *Mstn *gene appear to be resistant to skeletal muscle loss following transverse aortic constriction [30]. Hence, if this same mechanism is responsible for the development of cardiac cachexia in humans, blocking MSTN signalling in this disease context has the potential to target the actual root cause of skeletal muscle wasting, which has been shown to be a significant risk factor for mortality in patients with heart failure [31].

On the other hand, this question of underlying mechanism may be of purely academic interest if this therapeutic approach turns out to be effective in preserving muscle mass and reducing morbidity and mortality, regardless of the underlying cause of the cachexia. Indeed, the studies by Zhou *et al*. showing that the soluble receptor can improve survival of mice bearing certain tumours without directly perturbing the pathways that induce cancer or the consequent downstream activation of atrophy-signalling molecules secreted by the tumour provides compelling support for the notion that maintaining muscle mass is *per se *helpful in improving survival in otherwise cachectic conditions. An important question is how generalizable this beneficial effect on survival will turn out to be, not only with respect to cachexia induced by other types of cancers but also with respect to cachexia induced by other disease states. Similarly, it will be critical to determine whether the muscle anabolic capacity of agents like the soluble receptor varies depending on the physiological and disease context in which muscle loss is occurring. Given the extensive effort that is being directed by both the academic and biotechnology/pharmaceutical research communities to develop and test agents capable of blocking the MSTN/activin signalling pathway, it seems likely that we will have answers to at least some of these questions in the near future.

## Abbreviations

ActRIIB: ACVR2B (activin receptor type IIB); C26: colon-26; MSTN: myostatin; TGF-β: transforming growth factor-β; TSA: trichostatin A.

## Competing interests

Under a licensing agreement among MetaMorphix, Inc (MMI), Pfizer Inc and the Johns Hopkins University, SJL is entitled to a share of royalty received by the University on sales of products related to MSTN. SJL and the University own MMI stock, which is subject to certain restrictions under University policy. SJL, who is the scientific founder of MMI, is a consultant to MMI on research areas discussed in this paper. The terms of these arrangements are being managed by the University in accordance with its conflict of interest policies. DJG is an employee of Novartis.
